# History of malignant neoplasia lessens oocyte developmental competence: a case-control study

**DOI:** 10.5935/1518-0557.20210113

**Published:** 2023

**Authors:** Yuichi Jinno, Hisako Tobai, Shiori Imai, Miho Omura, Makoto Takeuchi, Mitsuyo Yoshida, Noriko Yano, Miki Goto, Takahide Arimoto

**Affiliations:** 1 Department of Obstetrics and Gynecology, Toranomon Hospital, Tokyo, Japan

**Keywords:** assisted reproductive technology, oocyte developmental competence, fertility preservation, history of cancer

## Abstract

**Objective:**

We investigated how history of malignant neoplasia affected oocyte developmental competence.

**Methods:**

Fifty-two cycles of assisted reproductive technology (ART) in women with a history of malignant disease (case group) were compared with fifty-two matched cycles of ART in women with no cancer history (control group). Propensity score matching involving age and body mass index was used to select controls. Oocyte developmental competence and rates of pregnancy and livebirth were compared as main outcomes. To investigate whether the cancer itself had affected oocyte developmental competence, this outcome variable was compared between case cycles with and without cancer surgical histories.

**Results:**

Numbers of fertilized oocytes (FO), cleaving embryos (CE), and superior CE (SCE) were significantly lower in cases than controls. Rates of fertilization and of development to SCE from retrieved oocytes (RO), FO, or CE also were lower in cases than controls (63, 25, 39, and 43% vs. 72, 36, 50, and 55%, respectively). Cases had significantly lower rates of clinical pregnancy and livebirth per embryo transfer than controls (7.6 and 1.5% vs. 20.4 and 14.0%). Rates of development to SCE from RO, FO, and CE showed no significance for differences between cases with and without cancer operations (22, 37, and 40% vs. 31, 42, and 49%).

**Conclusions:**

A woman's history of malignant neoplasia was associated with decreased oocyte developmental competence, possibly related to patient's background factors predisposing to tumor.

## INTRODUCTION

Cancer occurs all too frequently in young adults. For example, over 70,000 persons between 15 and 39 years old are diagnosed with malignant neoplasms each year in the US (Coccia *et al.,* 2014). In one study of cancer patients of reproductive age, 47 to 63% desired to have children after tumor diagnosis and treatment (Letourneau *et al.,* 2012). Embryo or oocyte cryopreservation before cancer treatment as fertility preservation is recommended by American and European oncologic societies (Loren *et al*., 2013; Peccatori *et al*., 2013).

Because embryo cryopreservation requires a committed sexual partner or a sperm donor, and may encounter social or religious objections in some countries and communities, oocyte cryopreservation is performed and investigated more often. In addition, a smaller fraction among women with a cancer history ultimately thaw their cryopreserved oocytes for childbearing than among women with no cancer history (Cobo *et al.,* 2018). For these reasons, the developmental competence of oocytes and pregnancy rates have not been investigated thoroughly for cryopreserved oocytes from patients with a history of malignant disease. Most previous studies have focused on differences in numbers of oocytes obtained for cryopreservation between women with and without a cancer diagnosis (Lekovich *et al.,* 2016; Klock *et al.,* 2010; Pal *et al.,* 1998; Quintero *et al.,* 2010; Robertson *et al.,* 2011; Quinn *et al.,* 2017; Almog *et al.,* 2012; Oktay *et al.,* 2006; Knopman *et al.,* 2009), rather than oocyte developmental potential or pregnancy rates (Cobo *et al.,* 2018; Pal *et al.,* 1998; Quintero *et al.,* 2010; Almog *et al.,* 2012; Oktay *et al.,* 2006). Furthermore, for both cryopreserved and fresh oocytes, the influence of a cancer history on assisted reproduction outcome is still unclear.

Some past studies suggested that malignant neoplasm could negatively affect fertility. A large cohort study of cancer survivor in Scotland showed a negative impact of cancer on subsequent achievement of pregnancy (Anderson *et al.,* 2018). In addition, concentrations of anti-Mullerian hormone have been found to be low in patients with cancer even before it is treated (Harzif *et al.,* 2019). Thus, patients with malignant neoplasia might have decreased ovarian reserve and potential for fertility even before beginning cancer treatment. However, whether the cause of these deficits lies in the tumor itself or in background factors predisposing to tumor.

To clarify effects of malignant tumors on oocyte developmental competence, we compared fertilization, embryonic development, and pregnancy rate between patients with and without a medical history of malignant tumors. In sub-analyses we investigated whether the malignant tumors themselves affected oocyte developmental competence.

## MATERIALS AND METHODS

### Study population

**Figure 1:**
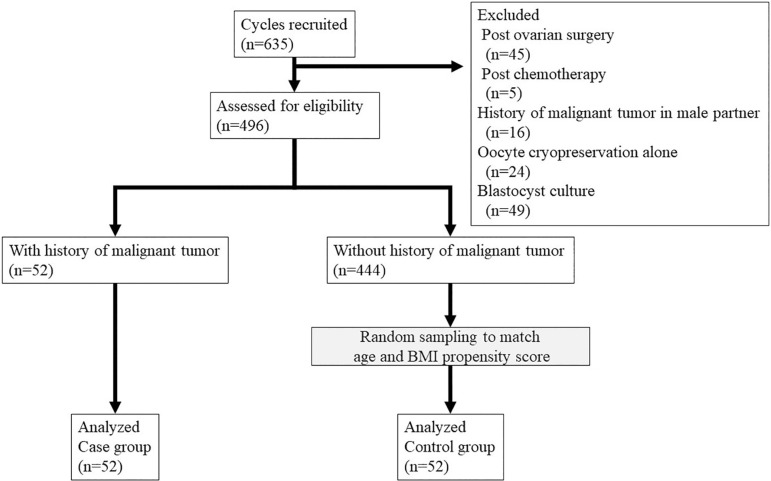
Study population enrollment flow chart. Among 635 cycles of ART performed at Toranomon Hospital from 2010 to 2019, 139 cycles were excluded because of previous ovarian surgery, previous chemotherapy, history of malignant tumor in male partner, oocyte cryopreservation, or blastocyst culture. The remaining 496 cycles represented our study population. Fifty-two cycles where women had a history of malignant tumor made up the case group. The 52 cycles in the control group were randomly chosen from the non-cancer study population by computer matching of propensity scores involving age and body mass index.

A case-control study was conducted starting with data from a total of 635 cycles of oocyte retrieval performed at Toranomon Hospital in Tokyo from January 2010 to December 2019. Since blastocyst cultures were carried out in only 49 cycles during this period, these were excluded from analysis. Ninety cycles were excluded because of previous ovarian surgery (45 cycles), previous chemotherapy (5 cycles), history of malignant tumor in male partner (16 cycles), or oocyte cryopreservation alone (24 cycles). The remaining 496 cycles represented our study population. The case group was defined as oocyte retrievals from women with a history of malignant neoplasia (52 cycles). From remaining the 444 cycles involving no such history, 52 control group cycles were randomly selected according to propensity score adjustment based on age and body mass index (BMI) (Fig. 1).

### Design of the case-control study

Oocytes were retrieved after stimulation with gonadotropin and gonadotropin releasing hormone (GnRH) agonist or antagonist. Fourteen cycles in the case group were stimulated without gonadotropin because the patient's breast cancer showed high hormonal receptivity based on receptor testing. *In vitro* fertilization (IVF) or intracytoplasmic sperm injection (ICSI) was selected according to condition of the semen. Fertilization was confirmed on the day after IVF/ICSI. Fresh embryo transfer (ET) or embryo cryopreservation was performed 2 or 3 days after oocyte retrieval. We defined superior cleaving embryos (SCE) as Grade 1 or 2 according to Veeck's criteria (Scott *et al.*, 1991). Pregnancy was defined as concentration of serum hCG beta subunit exceeding 50 IU/L at 14 days after embryo transfer. Clinical pregnancy was defined as detection of a gestational sac by transvaginal ultrasonography. Ongoing pregnancy was defined by detection of a fetal heartbeat.

Oocyte developmental competence and rates of pregnancy and live birth were compared between case and control groups as the main outcome measures of the study.

### Sub-analyses

Among the 52 cycles in the case group, the patient's history in 25 cycles involved breast cancer (48%); 9 cycles, cancer of the uterine corpus (17%); 2 cycles, cancer of the uterine cervix (4%); 9 cycles, cancer of the thyroid (17%); 3 cycles, gastrointestinal cancer (6%); and 4 cycles, lymphoma or leukemia (8%). The 25 cycles involving breast cancer included stage 0 in 1 cycle (4%), stage 1 in 20 cycles (80%), and stage 2 in 4 cycles (16%). To analyze the influence of breast cancer stage, we compared oocyte developmental competence between breast cancer stages in those 25 cycles (BC group). Next, to analyze the reproductive influence of malignant tumors apart from their treatment, we divided the case group into subgroups and compared oocyte developmental competence between cycles with and without surgical resections of malignant tumors (S+ *vs.* S-, n=33 and 19, respectively).

### Statistical analysis

Data were analyzed using IMB SPSS (Statistical Program for Social Sciences) software version 27. Student's t tests were used for normally distributed data. For non-normal distributions of data, the Mann-Whitney U test was used for bivariate comparisons, the Kruskal-Wallis test for comparisons of 3 or more groups, and the Bonferroni test as the post hoc test. Chi-squared tests and Fisher's exact test were used to compare ratios. Spearman's correlation coefficient was used for non-parametric data. *P* values below 0.05 was considered to indicate statistical significance. Results are presented as the mean ± standard deviation (SD).

### Ethical approval

This study was conducted with the approval of the Research Ethics Review Committee at the Toranomon Hospital. We also provided for patient optouts from the research plan according to the instructions of the Research Ethics Review Committee. 

## RESULTS

### Profiles of case-control study

No significant differences were evident between case and control groups for such general features as age (38.5±4.1 *vs.* 39.4±3.5), BMI (20.4±3.2 *vs.* 20.9±2.2), and basal endocrine profile values, except that baseline luteinizing hormone was significantly higher in the case than the control groups although the case group remained within the normal range (4.13±2.42 *vs.* 3.12±1.44; Table 1).

**Table 1:** Characteristics of case and control groups.

	Control group (n=52)	Case group (n=52)
Age (years)	39.4±3.5	38.5±4.1
BMI^a^ (kg/m^2^)	20.9±2.2	20.4±3.2
Baseline FSH^a^ (mIU/ml)	9.01±4.13	8.24 ± 2.53
Baseline LH^a^ (mIU/ml)	3.12±1.44	4.13±2.42^b^
Baseline E_2_^a^ (pg/ml)	57.2±35.4	55.6±40.5
Baseline T^a^	23.0±10.9	21.6±8.3
Baseline PRL^a^	15.7±8.9	17.7±11.2
HOMA-IR^a^	1.21±0.47	1.21±0.63

a BMI, body mass index; FSH, follicle-stimulating hormone; LH: luteinizing hormone; E_2_, estradiol; T, testosterone; PRL, prolactin; HOMA-IR, homeostasis model assessment - insulin resistance.

b *p*<0.05 *vs*. control, unpaired t test.

### Main outcomes of the case-control study

Values for FO, CE, and SCE were significantly lower in the case group than the control group (2.8±2.5, 2.5±2.2, and 1.1±1.2 *vs.* 3.8±2.6, 3.4±2.5, and 1.9±1.6; Table 2). Values for RO tended to be lower in the case group than in the control group (4.4±4.2 *vs.* 5.2±3.5; *p*=0.060, Mann-Whitney U test). The rate of fertilization was significantly lower in the case group than the control group (63% *vs.* 72%; Table 2). The case group had significantly lower rates of development to SCE from any of RO, FO, or CE than the control group (25, 39, and 43% *vs.* 36, 50, and 55%; *p*<0.05, Chi-squared test; odds ratios [OR], 0.578, 0.630, and 0.610; confidence intervals [CI], 0.392-0.853, 0.407-0.974, and 0.387-0.962, respectively). Correlation between age and number of RO, FO, CE, and SCE in case and control groups are shown in Table 3. Significant negative correlations were evident between age and number of RO and FO in the control group (R= -0.299 and -0.274), while no significant correlations were observed in the case group (R=-0.060 and -0.024). No significant correlations were present between age and number of CE or SCE in either group. Clinical outcomes are included in Table 2. The case group had significantly lower rates of clinical pregnancy and live birth per ET than the control group (respectively 7.6 and 1.5% *vs.* 20.4 and 14.0%; *p*<0.05, Chi-squared test; OR, 0.319 and 0.095; CI, 0.117-0.877 and 0.016-0.585).

**Table 2:** Developmental potentials of oocytes and clinical outcomes of embryo transfer in case and control groups.

	Control group		Case group		OR^a^	CI^a^
Numbers of RO^a^	5.2±3.5		4.4±4.2			
Numbers of FO^a^	3.8±2.6		2.8±2.5^b^			
Numbers of CE^a^	3.4±2.5		2.5±2.2^b^			
Numbers of SCE^a^	1.9±1.6		1.1±1.2^c^			
Fertilization rate (total numbers of FO/ total numbers of RO)	72%	(195/271)	63%^d^	(144/227)	0.676	0.464-0.986
Development rate to SCE from RO (total numbers of SCE/ total numbers of RO)	36%	(98/271)	25%^e^	(56/227)	0.578	0.392-0.853
Development rate to SCE from FO (total numbers of SCE/ total numbers of FO)	50%	(98/195)	39%^d^	(56/144)	0.630	0.407-0.974
Development rate to SCE from CE (total numbers of SCE/ total numbers of CE)	55%	(98/177)	43%^d^	(56/130)	0.610	0.387-0.962
Cycles of ET^a^ (cryopreserved + fresh)	93 cycles	66 cycles				
Embryos transferred per ET cycle	1.5±0.7		1.3±0.5			
% Clinical pregnancies per ET cycle	20.4%	19/93	7.6%^d^	5/66	0.319	0.117-0.877
% Ongoing pregnancies per ET cycle	15.1%	14/93	7.6%	5/66		
% Live deliveries per ET cycle	14.0%	13/93	1.5%^d^	1/66	0.095	0.016-0.585

a RO, retrieved oocytes; FO, fertilized oocytes; CE, cleaving embryos; SCE, superior cleaving embryos; ET, embryo transfer; OR, odds ratio; CI, confidence interval.

b *p*<0.05 *vs*. control group, Mann-Whitney U test

c *p*<0.01 *vs*. control group, Mann-Whitney U test

d *p*<0.05 *vs*. control group, Chi-squared test

e *p*<0.01 *vs*. control group, Chi-squared test

**Table 3:** Correlations between age and numbers of RO, FO, CE, or SCE in case and control groups.

	Case group	Control group
Number of ART cycles	52	52
Age...		
*vs*. number of RO^a^	R=-0.060	R=-0.299^b^
*vs*. number of FO^a^	R=-0.024	R=-0.274^b^
*vs*. number of CE^a^	R=-0.013	R=-0.231
*vs*. number of SCE^a^	R=0.200	R=-0.240

a RO, retrieved oocytes; FO, fertilized oocytes; CE, cleaving embryos; SCE, superior cleaving embryos.

b *p*<0.05, Spearman’s correlation coefficient.

### Results of sub-analyses

No significant differences in numbers of RO, FO, CE, and SCE were evident among stages 0, 1, and 2 of breast cancer (respectively 0±0, 0±0, 0±0, and 0±0 in stage 0; 3.2±4.5, 2.2±1.9, 2.1±1.8, and 1.1±1.3 in stage 1; and 3.8±4.3, 2.8±3.8, 2.8±2.0, and 1.3±1.5 in stage 2; Kruskal-Wallis test). No significant differences were evident in general characteristics between S+ and S- groups, except that baseline estradiol was significantly higher in the S+ group (63.8±46.4 *vs.* 42.8±23.4, Table 4). No significant difference was noted in numbers of RO, FO, CE, or SCE between S+ and S- groups, nor in rates of development to SCE (Table 5).

**Table 4:** Patient profiles for sub analysis of groups with (S+) and without (S-) cancer surgery.

	S+ group (n=33)	S- group (n=19)
Age (years)	38.3±2.9	38.8±5.7
BMI^a^ (kg/m^2^)	20.4±3.7	20.4±2.1
Baseline FSH^a^ (mIU/ml)	7.94±2.15	8.75±3.08
Baseline LH^a^ (mIU/ml)	3.70±1.71	4.88±3.24
Baseline E_2_^a^ (pg/ml)	63.8±46.4	42.8±23.4^b^
Baseline T^a^	21.2±8.37	22.2±8.35
Baseline PRL^a^	16.9±12.2	19.2±9.2
HOMA-IR^a^	1.11±0.51	1.52±0.86

a BMI, body mass index; FSH, follicle-stimulating hormone; LH, luteinizing hormone; E_2_, estradiol; T, testosterone; PRL, prolactin; HOMA-IR, homeostasis model assessment - insulin resistance.

b *p*<0.05 *vs*. control, unpaired t test.

**Table 5:** Fertilization and embryonic development potentials of oocytes from patient with (S+) and without (S-) cancer surgerys.

	S+ group (n=33)		S- group (n=19)	
Numbers of RO^a^	4.7±4.8		3.7±2.6	
Numbers of FO^a^	2.8±2.7		2.8±2.3	
Numbers of CE^a^	2.6±2.4		2.4±1.9	
Numbers of SCE^a^	1.0±1.2		1.2±1.0	
Fertilization rate (total numbers of FO/ total numbers of RO)	59%	(94/160)	75%^b^	(50/67)
Development rate to SCE from RO (total numbers of SCE/ total numbers of RO)	22%	(35/160)	31%	(21/67)
Development rate to SCE from FO (total numbers of SCE/ total numbers of FO)	37%	(35/94)	42%	(21/50)
Development rate to SCE from CE (total numbers of SCE/ total numbers of CE)	40%	(35/82)	49%	(21/43)

a RO, retrieved oocytes; FO, fertilized oocytes; CE, cleaving embryos; SCE, superior cleaving embryos.

b *p*<0.05 *vs*. S- group, Chi-squared test/Fisher’s exact test; odds ratio. 0.484; 95% confidence intervals, 0.258-0.908.

## DISCUSSION

This study investigated whether a history of malignant tumor affected oocyte developmental competence. Folliculogenesis was analyzed quantitatively by number of RO and qualitatively by oocyte developmental competence. The rates of development to SCE from RO, FO, and CE, which reflect oocyte developmental competence, all were significantly lower in the case group than the control group. The proportion of SCE among CE also was significantly lower in the case group. The number of RO was less in the case than the control group, but only at a borderline significance (*p*=0.06). Age and the number of RO showed a significant negative correlation in the control group but not in the case group, suggesting that ovarian dysfunction in the case group already was compromised, observing any effect of aging. FO, CE, and SCE, reflecting effects of both RO and oocyte developmental competence, again were significantly decreased in the case group. Altogether, our results indicate that women with a history of malignant disease are likely to have impaired folliculogenesis, sharply evident in terms of oocyte developmental competence and somewhat but less marked for number of RO.

Previous studies have shown conflicting results for number of RO, showing significant cancer related decreases in number of RO in some reports (Lekovich *et al.,* 2016; Klock *et al.,* 2010) but not others (Pal *et al.,* 1998; Quintero *et al.,* 2010; Robertson *et al.,* 2011; Quinn *et al.,* 2017; Almog *et al.*, 2012; Oktay *et al.,* 2006; Knopman *et al.,* 2009). Five of these previous studies (Lekovich *et al.,* 2016; Pal *et al.,* 1998; Quintero *et al.,* 2010; Almog *et al.,* 2012; Oktay *et al.,* 2006) considered fertilization rates, with 1 showing a significant decrease (Pal *et al.*, 1998) but 4 finding no difference. None of these studies examined embryonic development beyond fertilization. Patients in these studies were in their early 30s, which is considerably younger than the mean age of 39 years in our study. This age-related difference in influence of malignant disease on RO and fertilization rate might reflect a greater ovarian reserve in younger women.

In sub-analyses, no significant difference in oocyte developmental competence was evident between ART carried out after resection of malignant tumors and ART without prior tumor resection. In addition, we found no significant difference in oocyte development related to stage of breast cancer. These results suggest that impaired oocyte developmental competence in patients with a history of malignant tumor might not have resulted from the tumors, but possibly from background factors shared by cancer development and infertility. Insulin resistance which is involved in the mechanism underlying polycystic ovary syndrome (PCOS), a common disease underlying some case of infertility also has been suspected as a contributor to carcinogenesis associated with obesity (Wolin *et al.*, 2010). Reactive oxygen species similarly have been linked to progression of malignant tumors (Liou & Storz, 2010; Aggarwal *et al.,* 2019), ovulation disorders, decreased sperm function, and damage to embryos (Lu *et al.,* 2018). Reactive oxygen species also have been implicated in development of PCOS (Mohammadi, 2019) and endometriosis (Máté *et al.,* 2018). Relationships between advanced glycation end-products (AGE) and infertility (Jinno *et al.,* 2011), and between AGE and carcinogenesis (Walter *et al.*, 2019; Healey *et al.,* 2019) have been suggested. Thus, risk factors for malignant tumors and infertility often appear to overlap.

Because embryo cryopreservation for fertility preservation has encountered various difficulties, oocyte cryopreservation is more often performed and investigated at present. Further, fewer women with malignant tumors have been found to actually thaw their cryopreserved oocytes for childbearing than women who had undergone oocyte cryopreservation for other reasons than cancer (Cobo *et al.,* 2018). Our present investigation of oocyte cryopreservation in patients with a history of cancer showed significantly decreased rates of oocyte development and pregnancy, so women with malignant tumors might need to be warned about a higher risk for infertility despite ART. Further, when infertility is encountered, shared background factors might pose higher risk of malignant tumors later in life.
